# Effects of various exercise interventions for postpartum stress urinary incontinence: a systematic review and network meta-analysis

**DOI:** 10.3389/fmed.2026.1795125

**Published:** 2026-04-30

**Authors:** Yujia Yin, Jiawen Guo, Yating Wang, Huadong Li, Yingjie Qiao

**Affiliations:** 1 School of Acupuncture-Moxibustion and Tuina, Shandong University of Traditional Chinese Medicine, Jinan, Shandong, China; 2 Department of Tuina, The Affiliated Hospital of Shandong University of Traditional Chinese Medicine, Jinan, China

**Keywords:** exercise therapy, network meta-analysis, pelvic floor muscle training, postpartum, stress urinary incontinence

## Abstract

**Background:**

Exercise therapy is the first-line treatment for postpartum stress urinary incontinence (PSUI); however, the optimal regimen remains unclear.

**Objective:**

This study aimed to compare the effects of different exercise modalities on urinary leakage volume, quality of life, and pelvic floor muscle strength in PSUI patients using a network meta-analysis (NMA).

**Methods:**

Chinese and English databases were searched for randomized controlled trials (RCTs) from inception to September 2025. Two researchers independently screened studies, extracted data, and assessed the risk of bias. A network meta-analysis was performed using Stata 16.0, with treatment effects ranked by the surface under the cumulative ranking curve (SUCRA). The interventions reviewed included pelvic floor muscle training (PFMT) alone or combined with biofeedback, electrical stimulation, or vaginal weights, as well as aerobic exercise and traditional exercises. Treatment effects were expressed as standardized mean differences (SMDs) or odds ratios (ORs) with 95% confidence intervals (CIs).

**Results:**

A total of 49 RCTs involving 5,432 patients were included. SUCRA ranking indicated that PFMT combined with vaginal weights yielded the highest efficacy in reducing objective urinary leakage (1-h pad test, 92.1%; SMD vs. conventional care = 4.10, 95% CI: 0.29 to 7.92). For improving the quality of life, as measured by the International Consultation on Incontinence Questionnaire-Short Form (ICIQ-SF), PFMT combined with biofeedback and electrical stimulation (SMD = −4.41, 95% CI: −8.31 to −0.52) and aerobic exercise demonstrated superiority. For enhancing pelvic floor muscle strength, PFMT combined with vaginal weights (OR = 3.97, 95% CI: 2.15 to 5.79) and PFMT combined with biofeedback and electrical stimulation (OR = 4.07, 95% CI: 2.14 to 5.99) showed the most pronounced effects compared with conventional care.

**Conclusion:**

Among the various interventions, PFMT combined with vaginal weights is the most effective for reducing objective leakage, PFMT combined with biofeedback and electrical stimulation is the most effective for enhancing muscle strength, and aerobic exercise is the most effective for improving quality of life. To further validate these conclusions, large-scale, high-quality RCTs are needed.

**Systematic review registration:**

https://www.crd.york.ac.uk/prospero/, identifier CRD420251249264.

## 1 Introduction

Postpartum stress urinary incontinence (PSUI) is defined as involuntary urine leakage during activities that increase abdominal pressure, such as coughing, sneezing, or exercise. It is a common pelvic floor disorder affecting postpartum women. Its occurrence is often associated with damage to pelvic floor support structures during pregnancy and childbirth, including injury to pelvic floor muscles, fascia, and ligaments, leading to inadequate urethral support and weakened sphincter function (1, 2). Surveys indicate that more than 30% of postpartum women in China are affected, experiencing not only local discomfort but also frequent anxiety, social barriers, and reduced quality of life (3). Due to diverse intervention combinations and inconsistent outcome measures, current evidence remains fragmented and inconclusive. Existing studies have primarily focused on comparing specific exercise types with conventional pelvic floor muscle training (PFMT), while lacking direct comparisons between different combination regimens. This lack of direct evidence makes the superiority of specific exercise protocols controversial. Therefore, identifying effective and scalable exercise approaches has become a clinical priority. Using a network meta-analysis (NMA), this study systematically evaluates the effects of different exercise modalities on urinary leakage, quality of life, and pelvic floor muscle strength to provide evidence-based guidance for clinical decision-making.

## 2 Methods

To begin with, we submitted our application to the International Prospective Register of Systematic Reviews (PROSPERO) under registration number CRD420251249264. Then, this NMA was conducted in accordance with the guidelines provided by the Preferred Reporting Items for Systematic Review and Meta-Analyses (PRISMA) 2020 (4) and the Cochrane Handbook for Systematic Reviews of Interventions (5).

### 2.1 Eligibility criteria

The eligibility criteria were as follows:

1. The study type included randomized controlled trials (RCTs).
2. The study population included patients clinically diagnosed with PSUI. Diagnosis was based on the core symptom of involuntary urine leakage during increases in intra-abdominal pressure (e.g., sneezing, coughing, laughing, or exercise). Confirmation was obtained through validated questionnaires, such as the International Consultation on Incontinence Questionnaire-Short Form (ICIQ-SF), or objective tests (e.g., the 1-h pad test). There were no restrictions regarding parity, mode of delivery, or time postpartum.
3. Interventions: The intervention group received exercise therapy, including PFMT alone or combined with biofeedback, electrical stimulation, or vaginal weights; aerobic exercise; and traditional exercises. PFMT involved voluntary pelvic floor contractions (e.g., 8–12 contractions/session, 2–3 times/day, 8–12 weeks). Biofeedback provided real-time electromyography (EMG) feedback; electrical stimulation used low-frequency current (20–50 Hz); and vaginal weights involved weighted cones (20–70 g, 10–20 min/session). Aerobic exercise was of moderate-intensity (e.g., brisk walking, 30–45 min/session, 3–5 times/week). Traditional exercises included Baduanjin and Tai Chi (30–60 min/session, 3–5 times/week). Biofeedback alone, electrical stimulation alone, and their combination served as active comparators. The control group received standard care, sham intervention, or no treatment. Supporting references are provided in Table 1 notes.
4. Outcomes: The primary outcome was the change in the 1-h pad test weight. The secondary outcomes included ICIQ-SF scores, pelvic floor muscle strength, and psychosocial indicators (e.g., scores for psychological factors, social impairment, or behavioral restriction). Pelvic floor muscle strength was assessed using the Oxford Grading System. “Effective” improvement in pelvic floor muscle strength was defined as restoration to Grade IV or V (e.g., the ability to sustain a contraction for ≥4 s), while “ineffective” improvement was defined as failure to achieve this standard.

**Table 1:** Basic characteristics of the included studies.

Study ID	Intervention		Sample size		Age (years)		Gestational age		Treatment course (weeks)	Outcome measure
	C	T	C	T	C	T	C	T		
Zhong et al. (2021) (18)	A	H	30	30	28.23 ± 8.92	27.97 ± 6.28	/	/	8 w	ICIQ-SF score and psychosocial outcomes
Hu (2023) (19)	K	H	45	45	28.17 ± 6.28	27.56 ± 6.11	/	/	12 w	ICIQ-SF score, psychosocial outcomes, and treatment success rate
He (2018) (20)	A	E	25	25	26.5 ± 4.3	25.6 ± 4.3	40.0 ± 3.3	39.6 ± 3.3	8 w	Pelvic floor muscle strength, psychosocial outcomes, and incontinence incidence
Qin et al. (2016) (21)	D	E	165	165	24.3 ± 3.8	23.5 ± 5.3	/	/	8 w	1-h pad test, pelvic floor muscle strength, treatment success rate, and incontinence severity
Xiao (2018) (22)	B	E	55	55	47.27 ± 10.54	47.13 ± 10.14	/	/	8 w	1-h pad test, pelvic floor muscle strength, leakage volume, incontinence severity, and treatment success rate
Sun (2018) (23)	B	C	43	43	27.2 ± 3.5	27.5 ± 3.4	38.6 ± 1.2	38.9 ± 1.2	16 w	Pelvic floor muscle strength, voiding function, and complications
Xu (2016) (24)	A	B	40	40	42.4 ± 5.4	43.2 ± 5.2	/	/	/	Psychosocial outcomes
Yun and Zhao (2023) (25)	J	I	36	36	30.25 ± 3.46	30.31 ± 3.54	/	/	2 w	ICIQ-SF score, pelvic floor muscle strength, psychosocial outcomes, and urodynamic parameters
Shi et al. (2017) (26)	B	F	48	48	54.05 ± 9.34	52.9 ± 7.68	/	/	12 w	Psychosocial outcomes
Qi (2020) (27)	A	C	30	30	26.59 ± 5.25	39.1 ± 1.2	38.7 ± 1.2	39.1 ± 1.2	8 w	Pelvic floor muscle strength and voiding function
Huang et al. (2011) (28)	A	B	48	48	/	/	/	/	8 w	1-h pad test and pelvic floor muscle strength
Wu (2013) (29)	A	B	44	44	28.3 ± 1.7	27.6 ± 1.5	25.4 ± 1.6	24.8 ± 1.7	8 w	1-h pad test and pelvic floor muscle strength
Duan et al. (2014) (30)	A	B	40	40	31.00 ± 4.00	30.00 ± 4.00	26.13 ± 1.55	26.41 ± 1.67	/	1-h pad test, leakage volume, pelvic floor muscle strength, and treatment success rate
Dai (2016) (31)	A	B	50	50	48.5 ± 2.3	48.5 ± 2.3	/	/	12 w	1-h pad test, ICIQ-SF score, and treatment success rate
Li et al. (2024) (32)	A	B	44	44	30.33 ± 1.83	30.22 ± 1.78	/	/	2 w	Psychosocial outcomes, voiding function, and EMG values
Liu et al. (2024) (33)	D	C	50	50	/	/	/	/	2 w	1-h pad test, ICIQ-SF score, pelvic floor muscle strength, psychosocial outcomes, and treatment success rate
Li (2020) (34)	A	B	102	102	31.2 ± 1.5	30.8 ± 1.9	/	/	12 w	Pelvic floor muscle strength and incontinence status
Pang et al. (2021) (35)	D	C	30	30	35.2 ± 2.2	36.1 ± 2.4	/	/	12 w	Pelvic floor muscle strength, incontinence status, voiding function, and treatment success rate
Zhang (2022) (36)	D	C	58	58	28.28 ± 3.13	28.04 ± 3.22	39.46 ± 1.14	39.41 ± 1.15	6 w	Pelvic floor muscle strength, psychosocial outcomes, and incontinence status
Wen et al. (2010) (37)	A	B	73	75	28.23 ± 7.92	28.17 ± 7.12	39.18 ± 1.45	38.38 ± 1.5	6 w	1-h pad test, pelvic floor muscle strength, and voiding function
Zhang (2020) (38)	A	B	35	35	52.77 ± 7.86	52.64 ± 7.56	/	/	/	ICIQ-SF score, incontinence episodes, incontinence volume, and treatment success rate
Zhou et al. (2017) (39)	A	B	51	50	52.74 ± 7.34	52.42 ± 7.06	/	/	12 w	ICIQ-SF score, incontinence episodes, leakage volume, incontinence severity, and treatment success rate
Qi et al. (2020) (40)	A	B	50	50	31.69 ± 4.65	30.24 ± 4.61	30.08 ± 1.39	30.11 ± 1.42	8 w	ICIQ-SF score, leakage volume, pelvic floor muscle strength (Type I/II), and urodynamic parameters
Qi et al. (2020) (40)	A	D	50	50	31.69 ± 4.65	31.06 ± 4.64	30.08 ± 1.39	31.06 ± 1.47	8 w	ICIQ-SF score, leakage volume, pelvic floor muscle strength (Type I/II), and urodynamic parameters
Huang (2011) (41)	A	B	27	27	26.51 ± 4.2	25.62 ± 4.4	37.9 ± 1.4	38.4 ± 1.3	8 w	1-h pad test, pelvic floor muscle strength, and voiding function
Wu (2013) (42)	A	B	30	30	28.57 ± 5.48	28.27 ± 5.28	38.9 ± 1.5	39.2 ± 1.4	8 w	1-h pad test, pelvic floor muscle strength, and voiding function
Yue (2016) (43)	A	B	30	30	26.51 ± 4.5	28.34 ± 5.21	38.8 ± 1.6	39.2 ± 1.4	8 w	Pelvic floor muscle strength and voiding function
Zhang et al. (2011) (44)	A	E	315	315	31.2 ± 1.6	31.16 ± 2.34	39.1 ± 1.1	38.6 ± 1.5	/	Pelvic floor muscle strength
Chen (2017) (45)	A	B	100	100	25.0 ± 1.5	27.0 ± 1.5	36.0 ± 3.5	37.0 ± 2.5	10 w	1-h pad test, pelvic floor muscle strength, and treatment success rate
Ye et al. (2015) (46)	A	B	30	30	26.70 ± 0.80	29.30 ± 1.20	38.58 ± 1.52	38.38 ± 1.50	12 w	1-h pad test, pelvic floor muscle strength, and voiding function
Sun et al. (2025) (47)	A	B	40	40	29.12 ± 1.86	28.96 ± 1.84	/	/	4 w	Pelvic floor muscle strength, incontinence status, and treatment success rate
Tang et al. (2017) (48)	A	B	59	59	45.3 ± 3.2	45.3 ± 3.2	/	/	24 w	ICIQ-SF score and pelvic floor muscle strength
Liu (2025) (49)	A	B	32	32	30.25 ± 8.46	30.25 ± 8.46	38.8 ± 1.42	38.8 ± 1.42	12 w	ICIQ-SF score, 1-h pad test, pelvic floor muscle strength, and psychosocial outcomes
Zhang. (2023) (50)	B	E	31	31	27.01 ± 0.79	26.86 ± 0.75	37.62 ± 1.35	37.57 ± 1.33	8 w	Psychosocial outcomes, pelvic floor muscle strength (Type I/II), and urodynamic parameters
Huo and Lei (2020) (51)	B	C	44	44	46.26 ± 2.55	45.98 ± 2.66	/	/	8 w	Pelvic floor muscle strength and treatment success rate
Wang (2017) (52)	A	C	30	30	44.06 ± 3.97	42.16 ± 3.75	/	/	6 w	ICIQ-SF score and treatment success rate
Ju (2014) (53)	G	B	30	30	29.37 ± 4.26	29.13 ± 4.31	39.07 ± 1.44	38.97 ± 1.30	8 w	1-h pad test and pelvic floor muscle strength
Xiao (2021) (54)	D	B	35	36	25.36 ± 1.02	25.41 ± 1.01	39.55 ± 0.63	39.61 ± 0.64	8 w	1-h pad test, pelvic floor muscle strength (Type I/II), and treatment success rate
Zhang (2017) (55)	G	B	40	40	29.14 ± 3.97	29.42 ± 4.33	38.64 ± 1.32	39.06 ± 1.36	8 w	1-h pad test, EMG values, and urodynamic parameters
Zheng et al. (2024) (56)	B	C	34	34	45.63 ± 6.45	42.32 ± 5.24	/	/	8 w	ICIQ-SF score, pelvic floor muscle strength, incontinence frequency, cystometry, and treatment success rate
Zhang (2021) (57)	B	G	43	43	39.81 ± 4.24	39.78 ± 4.19	/	/	12 w	1-h pad test, ICIQ-SF score, psychosocial outcomes, and treatment success rate
Peng (2019) (58)	B	G	50	50	40.3 ± 9.4	39.6 ± 9.2	/	/	12 w	Psychosocial outcomes, 1-h pad test, EMG values, and treatment success rate
Ye (2018) (59)	B	F	35	35	38.79 ± 12.71	40.25 ± 13.06	/	/	8 w	ICIQ-SF score, pelvic floor muscle strength, and urodynamic parameters
Tu et al. (2016) (60)	B	E	120	120	29.14 ± 7.87	30.25 ± 8.76	38.58 ± 1.51	38.55 ± 1.47	8 w	1-h pad test, pelvic floor muscle strength, and voiding function
Jiang et al. (2017) (61)	D	B	62	63	27.32 ± 0.81	27.32 ± 0.81	39.03 ± 1.74	39.03 ± 1.74	/	Pelvic floor muscle strength and treatment success rate
Li et al. (2013) (62)	A	F	100	100	27.52 ± 0.71	27.52 ± 0.71	/	/	/	Pelvic floor muscle strength and incontinence status
Zhang et al. (2022) (63)	B	H	45	45	49.11 ± 9.18	46.98 ± 9.19	/	/	4 w	ICIQ-SF score and treatment success rate
Li et al. (2012) (64)	A	B	40	40	29.2 ± 3.6	29.0 ± 3.7	38 ± 1.53	38.59 ± 1.53	24 w	1-h pad test, pelvic floor muscle strength, and voiding function
Hu et al. (2011) (65)	A	B	32	35	29.15 ± 6.82	29.15 ± 6.82	/	/	12 w	1-h pad test, pelvic floor muscle strength, and voiding function
Khorasani et al. (2020) (66)	A	B	40	40	30.25 ± 5.65	30.75 ± 5.09	/	/	12 w	ICIQ-SF score and pelvic floor muscle strength

Intervention codes: A: conventional care; B: PFMT alone; C: PFMT + biofeedback + electrical stimulation; D: biofeedback + electrical stimulation; E: PFMT + vaginal weights; F: aerobic exercise; G: PFMT + biofeedback; H: traditional exercises; I: PFMT + electrical stimulation; J: electrical stimulation alone; K: biofeedback alone; EMG, electromyography. For detailed descriptions of each intervention, see Section 2.1.

### 2.2 Search strategy

We systematically searched the following electronic databases from inception to September 2025: PubMed, Embase, the Cochrane Library, Web of Science, Chinese SinoMed, China National Knowledge Infrastructure (CNKI), Chinese VIP Database for Technical Periodicals (VIP), and Wanfang. A combination of Medical Subject Headings (MeSH) and free-text terms was used to retrieve relevant studies. The detailed search strategies for all databases are provided in Supplementary File 1.

### 2.3 Study selection

All search results were imported into NoteExpress 4.2 for automated and manual deduplication. Two reviewers independently screened the literature based on predefined eligibility criteria, first by titles and abstracts and then by full-text assessment of potentially eligible studies. Disagreements were resolved through discussion or, if necessary, by consulting a third reviewer. Using a standardized form, the same two reviewers independently extracted the following data: first author, publication year, participant characteristics (sample size, age, etc.), exercise intervention details (specific protocol, duration, etc.), and outcome measures. Data were cross-checked to ensure accuracy.

### 2.4 Risk of bias

We assessed the methodological quality of the included RCTs using the revised Cochrane Risk of Bias 2.0 tool (ROB 2.0) (5). This tool evaluates five domains: the randomization process, deviations from the intended interventions, missing outcome data, measurement of the outcome, and selection of the reported result. Each domain was rated as “low risk of bias,” “some concerns,” or “high risk of bias,” and an overall risk of bias judgment was derived for each study. The results were presented graphically to provide a comprehensive overview of the methodological quality across all included studies.

### 2.5 Statistical analysis

We performed an NMA within a frequentist framework using Stata 16.0. For continuous outcomes, we calculated standardized mean differences (SMDs) with 95% confidence intervals (CIs) to account for variations in measurement tools or units across studies. For the dichotomous outcome of pelvic floor muscle strength improvement, odds ratios (ORs) with 95% CIs were used. A network geometry plot was generated for each outcome to illustrate the available direct comparisons. Global inconsistency was assessed using design-by-treatment interaction models. A consistency model was adopted if no significant inconsistency was detected (*p* ≥ 0.05); otherwise, an inconsistency model was considered. The surface under the cumulative ranking curve (SUCRA) was used to rank the comparative effectiveness of the interventions. The results were summarized in league tables and forest plots. Potential publication bias was assessed using comparison-adjusted funnel plots.

### 2.6 Assessment of transitivity

The validity of the NMA depends on the transitivity assumption that patients in different studies are sufficiently similar to allow indirect comparisons. To assess this, we examined the distribution of potential effect modifiers across treatment nodes, including patient age, time since delivery, baseline incontinence severity, and study duration. These characteristics were broadly comparable across studies, supporting the plausibility of the transitivity assumption.

## 3 Results

The main findings for the primary outcomes are presented below. Extensive supplementary results, including network plots, detailed forest plots, ranking plots, league tables, and publication bias assessments for all outcome measures, are provided in Supplementary Files 2–7.

### 3.1 Literature search and study characteristics

**Figure 1:**
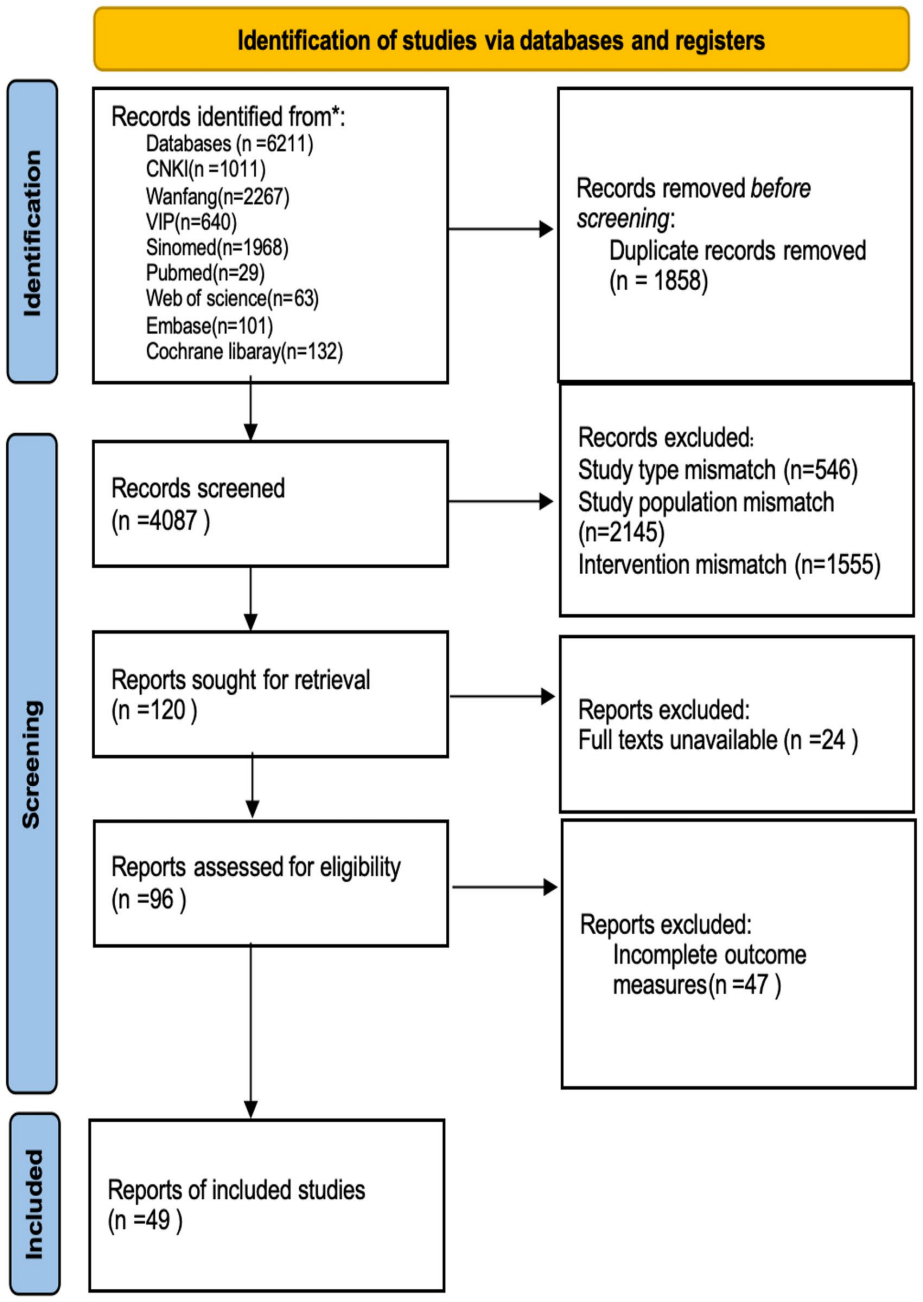
PRISMA flow diagram for study selection.

The initial search identified 6,221 publications. After screening titles, abstracts, and full texts, 49 RCTs involving 5,432 patients were included. Table 1 summarizes the basic characteristics of the included studies, and Figure 1 shows the literature screening process. The included studies varied in patient characteristics: time since delivery ranged from 6 weeks to 12 months postpartum, and incontinence severity ranged from mild to moderate based on reported pad test results or ICIQ-SF scores. However, inconsistent reporting of these variables across studies precluded subgroup analyses based on these factors.

### 3.2 Risk of bias of the included studies

**Figure 2:**
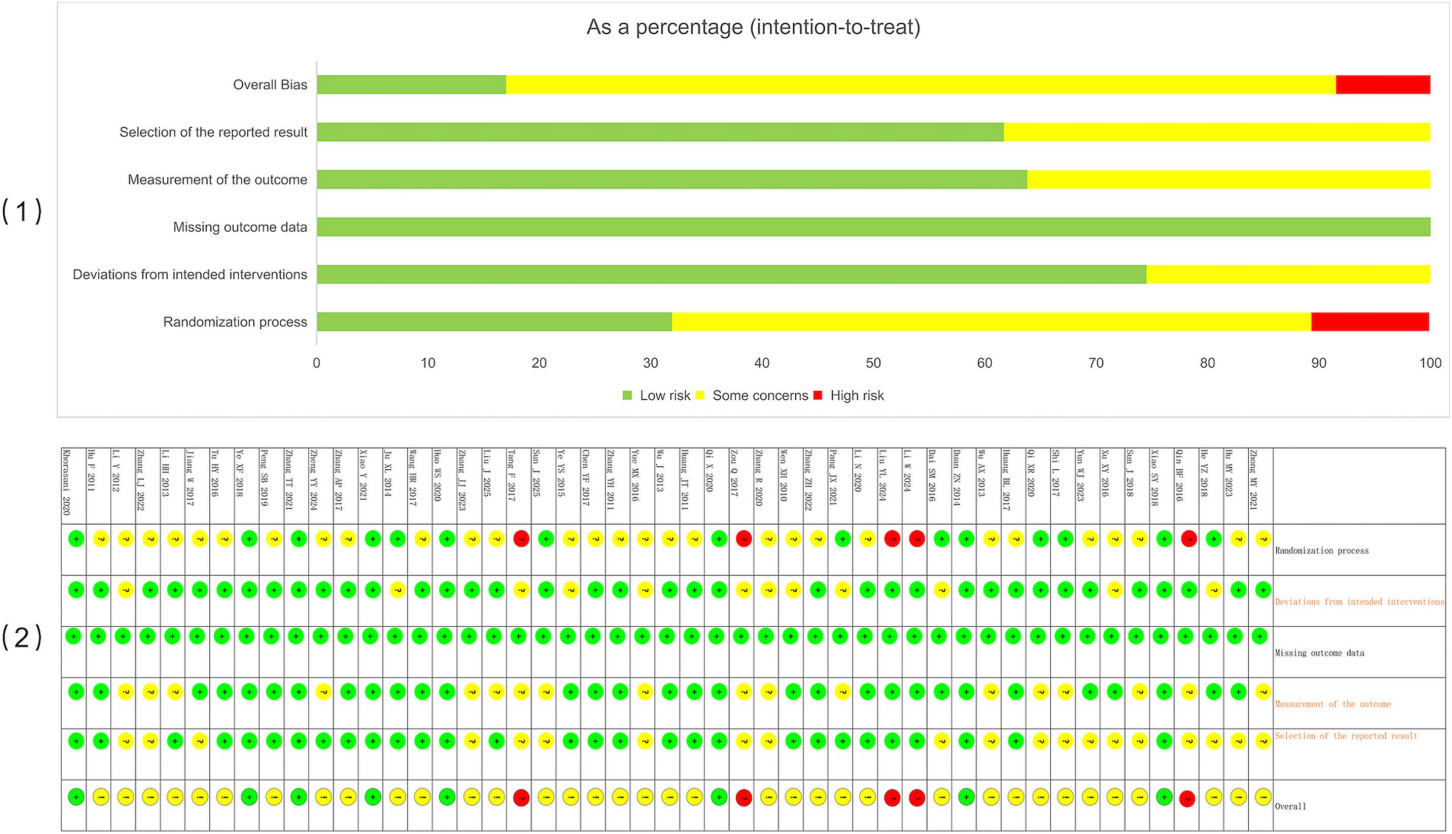
Risk of bias assessment. (1) Risk of bias for each included study; (2) overall summary of risk of bias across all studies.

The Cochrane ROB 2.0 tool was used to assess the risk of bias in the included studies. Of the 49 included RCTs, 8 studies were classified as having a low risk of bias, while the remaining studies were rated as having some concerns, primarily due to insufficient details on the randomization process, allocation concealment, and blinding. Overall, the methodological quality of the included studies was considered acceptable. A detailed summary of the risk of bias assessment is presented in Figure 2. The predominance of studies rated as “some concerns” for bias should be considered when interpreting the subsequent NMA results.

### 3.3 Network evidence relationships

**Figure 3:**
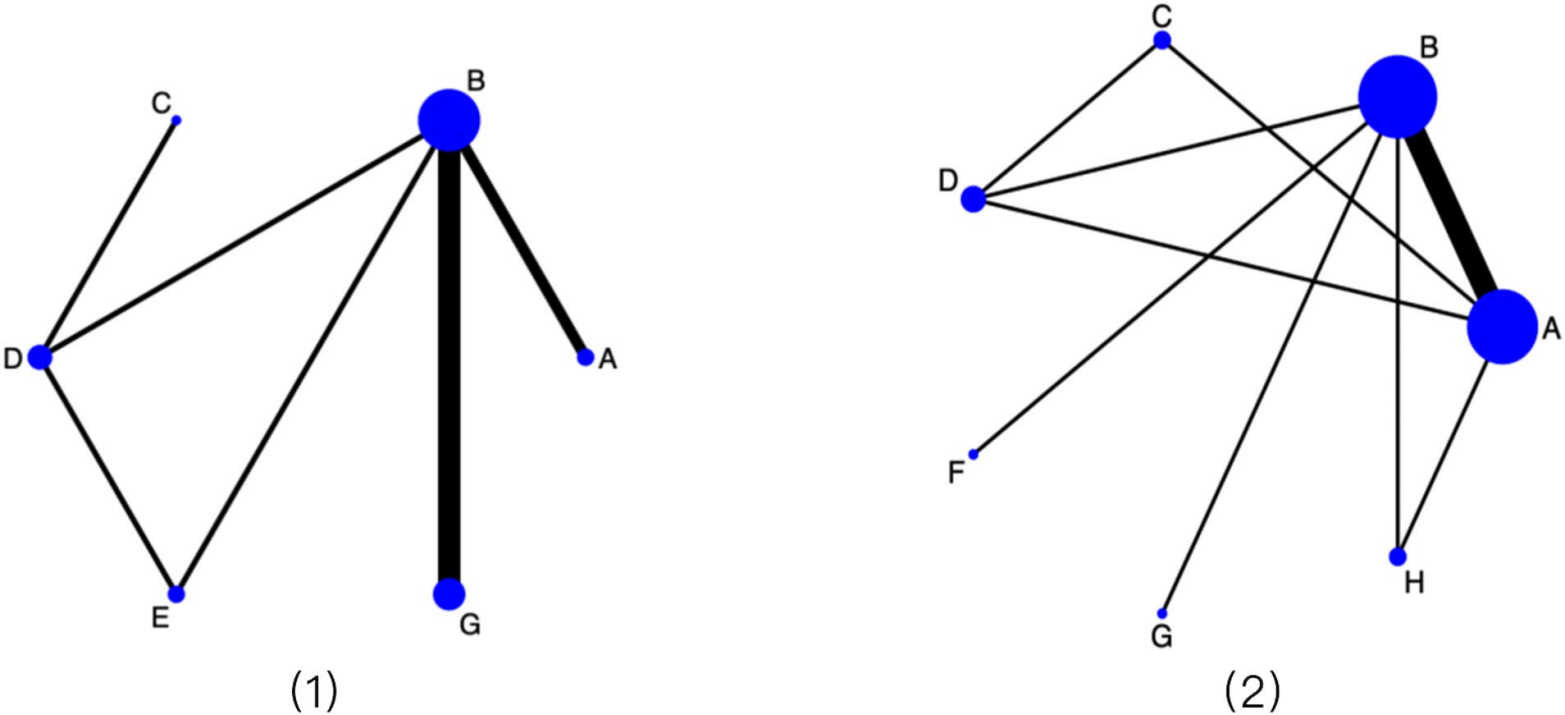
Network evidence diagram for primary outcomes. (1) 1-h pad test; (2) ICIQ-SF scores. Interventions are coded as follows: A: Conventional care; B: PFMT alone; C: PFMT + biofeedback + electrical stimulation; D: Biofeedback + electrical stimulation; E: PFMT + vaginal weights; F: Aerobic exercise; G: PFMT + biofeedback; H: Traditional exercises.

Network evidence plots for the primary outcomes illustrated that PFMT was the most frequently connected intervention within the network. It formed direct comparisons with most other exercise modalities, resulting in a well-connected, star-shaped network structure that was suitable for network meta-analysis. The network diagrams for the primary outcomes are presented in Figure 3.

### 3.4 Network meta-analysis results

#### 3.4.1 1-h pad test

**Figure 4:**
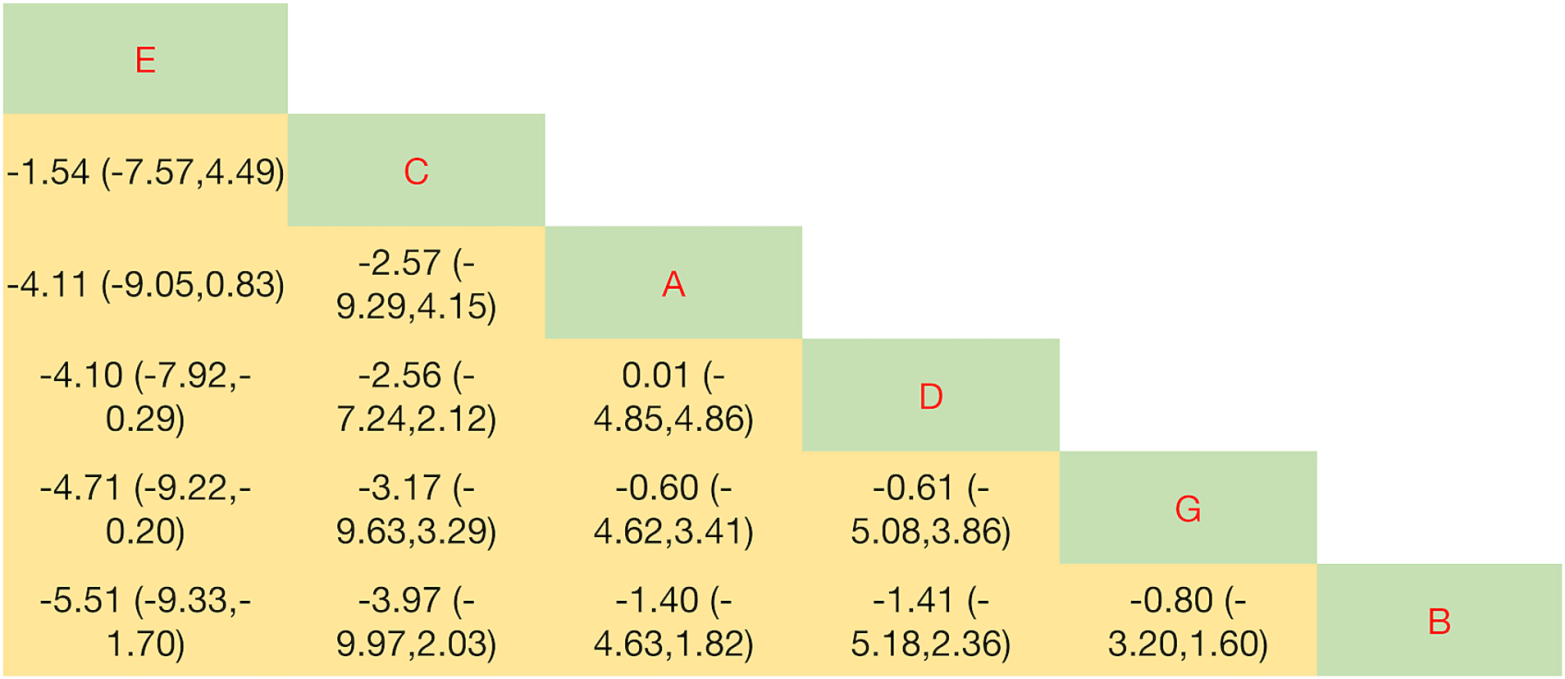
League table for 1-h pad test. Data are SMD (95% CI) for column vs. row. SMD < 0 favors column; SMD > 0 favors row. Interventions are coded as follows: A: conventional care; B: PFMT alone; C: PFMT + biofeedback + electrical stimulation; D: biofeedback + electrical stimulation; E: PFMT + vaginal weights; G: PFMT + biofeedback.

Significant differences in objective leakage volume were observed between five exercise interventions and conventional care, as shown in Figure 4. The NMA indicated that PFMT + vaginal weights had the highest probability of being the optimal intervention (SUCRA = 92.1%), followed by PFMT + biofeedback + electrical stimulation (73.3%), PFMT + biofeedback (44.1%), and biofeedback + electrical stimulation (40.7%). Biofeedback alone demonstrated the lowest efficacy (SUCRA = 15.9%). Compared with conventional care, PFMT + vaginal weights significantly reduced leakage volume (SMD = 4.10, 95% CI: 0.29 to 7.92). In direct comparisons, PFMT + vaginal weights were more effective than PFMT alone (SMD = 5.51, 95% CI: 1.70 to 9.33) and biofeedback + electrical stimulation (SMD = 4.71, 95% CI: 0.20 to 9.22). No other intervention was statistically superior to conventional care or to other comparators for this outcome. Complete pairwise comparison results are presented in the league table (Figure 4).

#### 3.4.2 ICIQ-SF scores

**Figure 5:**
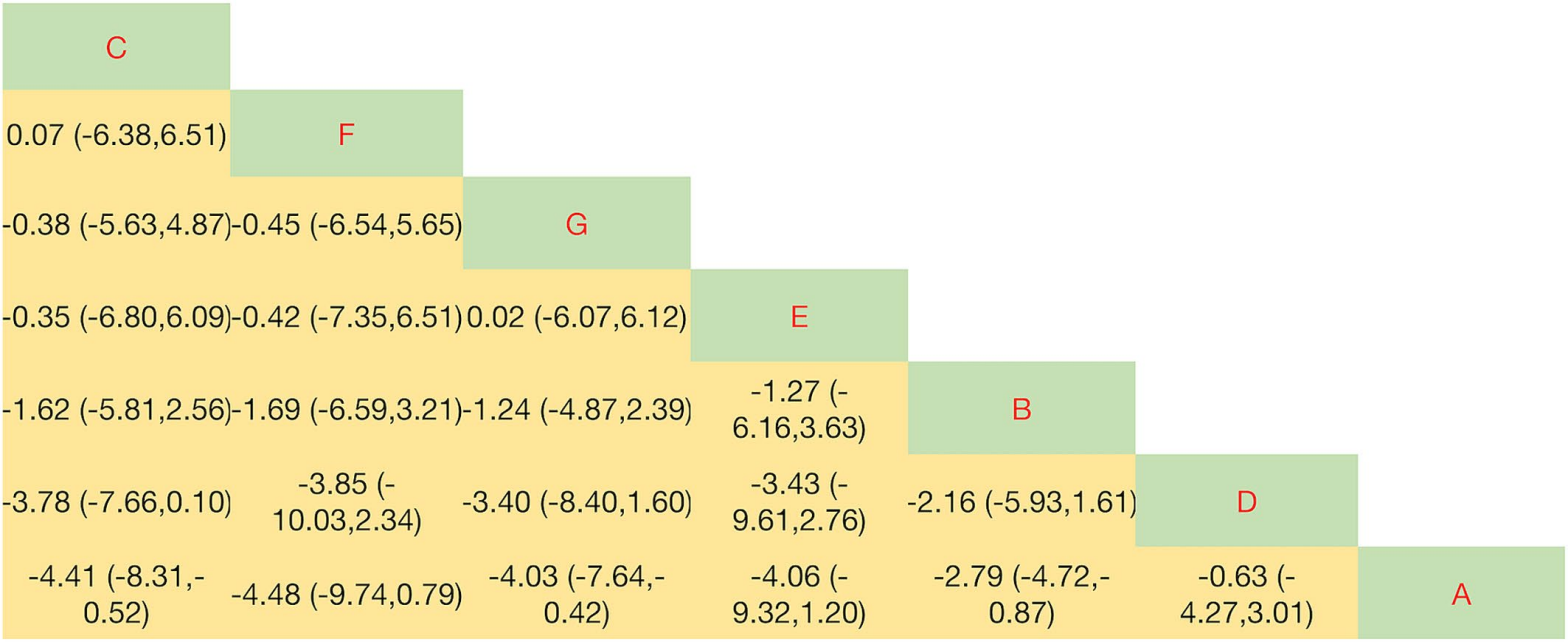
League table for ICIQ-SF scores. Data are SMD (95% CI) for column vs. row. SMD < 0 favors column; SMD > 0 favors row. Interventions are coded as follows: A: Conventional care; B: PFMT alone; C: PFMT + biofeedback + electrical stimulation; D: Biofeedback + electrical stimulation; E: PFMT + vaginal weights; F: Aerobic exercise; G: PFMT + biofeedback.

As illustrated in Figure 5, several exercise modalities improved quality of life compared with conventional care. SUCRA rankings suggested that PFMT + biofeedback + electrical stimulation had the highest probability of being the optimal intervention (SUCRA = 72.3%). This combination significantly improved ICIQ-SF scores compared with conventional care (SMD = −4.41, 95% CI: −8.31 to −0.52). Aerobic exercise (SUCRA = 67.0%) and traditional exercises (SUCRA = 30.4%) also ranked highly; however, their direct comparisons with conventional care did not reach statistical significance (SMD = −0.63, 95% CI: −4.27 to 3.01; and SMD = −4.48, 95% CI: −9.74 to 0.79, respectively). Complete pairwise comparison data are presented in Figure 5.

#### 3.4.3 Pelvic floor muscle strength improvement

**Figure 6:**
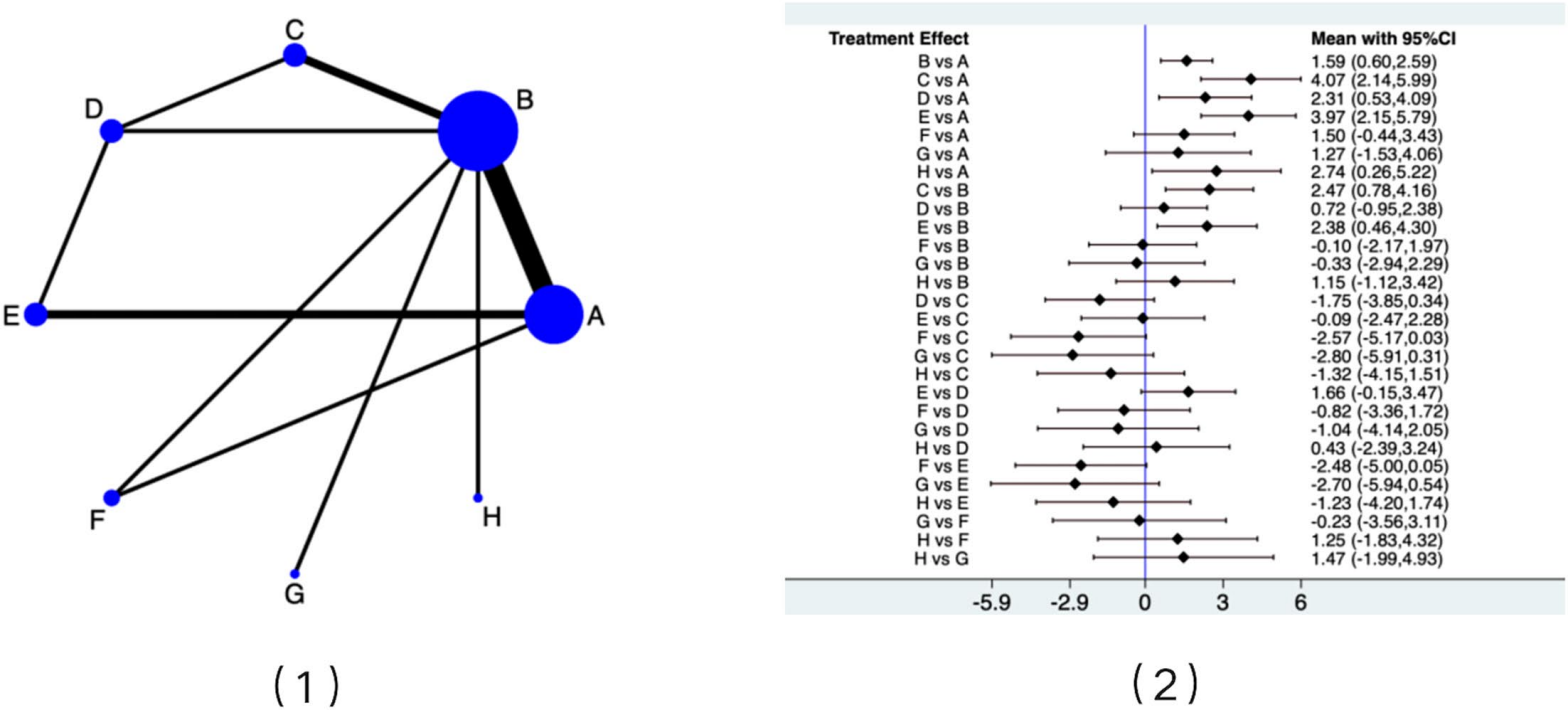
(1) Evidence network map for pelvic floor muscle strength improvement; (2) Forest plot of pelvic floor muscle strength improvement. Results are presented as odds ratios (ORs) with 95% confidence intervals. Interventions are coded as follows: A: Conventional care; B: PFMT alone; C: PFMT + biofeedback + electrical stimulation; D: Biofeedback + electrical stimulation; E: PFMT + vaginal weights; F: Aerobic exercise; G: PFMT + biofeedback; H: Traditional exercises.

Figure 6 presents the evidence network for pelvic floor muscle strength improvement. Based on SUCRA rankings, PFMT + vaginal weights and PFMT + biofeedback + electrical stimulation had the highest probability of being optimal interventions. Consistent with this ranking, the forest plot (Figure 6) showed that most exercise programs outperformed routine care. PFMT + vaginal weights (OR = 3.97, 95% CI: 2.15 to 5.79) and PFMT + biofeedback + electrical stimulation (OR = 4.07, 95% CI: 2.14 to 5.99) demonstrated the largest effect sizes. Improvements were also observed for PFMT alone (OR = 1.59, 95% CI: 0.60 to 2.59), biofeedback + electrical stimulation (OR = 2.31, 95% CI: 0.53 to 4.09), and traditional exercises (OR = 2.74, 95% CI: 0.26 to 5.22). In direct comparison, PFMT + vaginal weights were superior to PFMT alone (OR = 2.38, 95% CI: 0.46 to 4.30).

#### 3.4.4 Secondary psychosocial outcomes

The NMA revealed substantial inconsistency across evidence networks for psychological factors, social barriers, and behavioral limitations (all *p* < 0.05). Based on SUCRA rankings, interventions were ordered as follows: aerobic exercise, PFMT + biofeedback + electrical stimulation, traditional exercises, PFMT + vaginal weights, PFMT + biofeedback, biofeedback + electrical stimulation, PFMT, and routine postpartum care. Given the significant inconsistency, these findings should be interpreted with caution, and further high-quality studies are needed.

### 3.5 Publication bias

**Figure 7:**
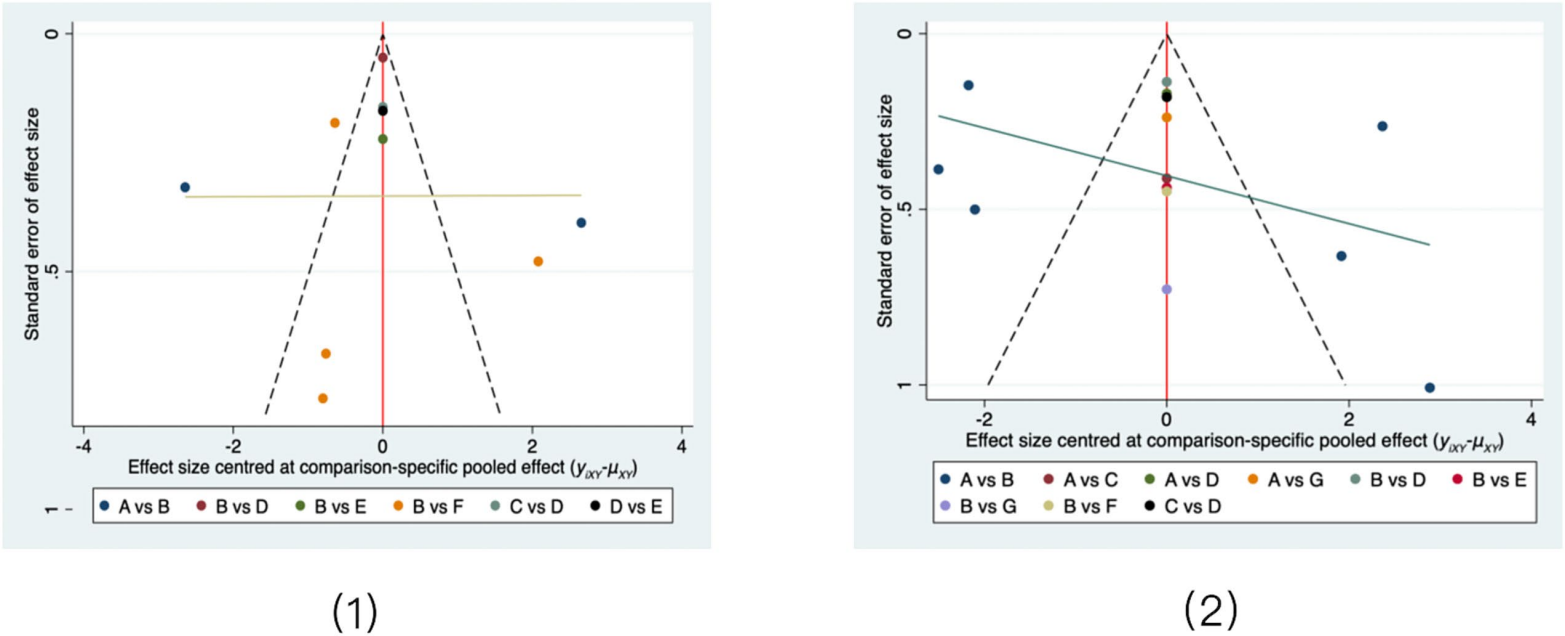
Comparison-adjusted funnel plots for primary outcomes. (1) 1-h pad test; (2) ICIQ-SF scores. Interventions are coded as follows: A: Conventional care; B: PFMT alone; C: PFMT + biofeedback + electrical stimulation; D: Biofeedback + electrical stimulation; E: PFMT + vaginal weights; F: Aerobic exercise; G: PFMT + biofeedback.

As shown in Figure 7, the comparison-adjusted funnel plots for the primary outcomes were broadly symmetrical, suggesting a low likelihood of publication bias. This visual assessment was further supported by Egger’s test (1-h pad test: *p* = 0.447; ICIQ-SF scores: *p* = 0.396), as detailed in Supplementary File 6. Funnel plots for secondary outcomes are presented in Supplementary File 7.

## 4 Discussion

### 4.1 Summary of the main findings

This NMA systematically compared the efficacy of 10 exercise interventions for postpartum stress urinary incontinence. The results indicated that different exercise modalities have different advantages in improving objective leakage volume, quality of life, and pelvic floor muscle strength. These differences can be explained by their underlying mechanisms of action.

### 4.2 PFMT combined therapy

Our findings suggest that PFMT combined with vaginal weights or PFMT combined with biofeedback and electrical stimulation may offer greater improvements in objective leakage volume and pelvic floor muscle strength compared with PFMT alone in patients with PSUI. This advantage may be attributed to synergistic physiological and neuromuscular effects rather than simple additive effects (6).

PFMT alone relies on patients’ proprioception, which can be weak and imprecise, making it difficult to ensure adequate training intensity and quality. Combining PFMT with vaginal weights introduces quantifiable physical load, shifting training from subjective sensation to goal-oriented progressive resistance. The vertical gravitational force of vaginal weights stimulates both Type I and Type II muscle fibers, potentially enhancing pelvic floor endurance and explosive strength. This may, in turn, support urethral structures and improve sphincter closure under stress (7).

Combining PFMT with biofeedback and electrical stimulation may facilitate neuromuscular functional remodeling through multiple pathways. Biofeedback translates electromyographic activity into visual signals, allowing real-time correction of contraction strategies and helping to address compensatory patterns caused by poor proprioception (8). Electrical stimulation activates motor neurons via low-frequency pulses, which may help maintain muscle function and support neural recovery (9, 10). Together, these components may form an integrated approach: electrical stimulation provides neural activation, biofeedback guides active learning and control, and PFMT reinforces strength and endurance. This combination may contribute to pelvic floor rehabilitation from spinal reflexes up to cortical control (11–13).

In summary, these combined interventions optimize PFMT by addressing biomechanical resistance and neurophysiological regulation. International guidelines, including those from the American Urological Association, recommend such combined approaches as first-line conservative treatments for SUI based on current evidence (14).

### 4.3 Aerobic exercise

This study found that aerobic exercise was associated with improvements in patients’ quality of life. Aerobic exercise primarily consists of moderate-intensity activities such as walking, jogging, and swimming. This finding is consistent with previous research (15).

Several mechanisms may explain this effect. Aerobic exercise improves systemic blood circulation and autonomic nervous system regulation, potentially reducing anxiety and tension at the whole-body level, which could enhance patients’ confidence in urinary control. Compared with PFMT alone, aerobic exercise showed advantages in improving life satisfaction and reducing social limitations associated with urinary incontinence. Additionally, aerobic exercise promotes the release of neurotransmitters such as endorphins, which may help improve mood. This is particularly relevant for postpartum women experiencing the psychological burden of urinary leakage (16).

### 4.4 Traditional exercises

Among patients with PSUI, commonly adopted exercise regimens include Tai Chi and Baduanjin. Our findings suggest that traditional exercises may offer certain advantages in improving quality of life compared with some combined exercise programs. This observation aligns with the broader trend of integrating traditional and complementary therapies in women’s health (17).

The mechanism of action may involve the integration of intention, breathing, and movement emphasized in traditional exercises. Through slow, continuous, and rhythmic movements, these practices regulate autonomic nervous system function, promote physical and mental relaxation, and reduce stress levels. This could be particularly beneficial for alleviating anxiety and social avoidance associated with urinary incontinence. Additionally, traditional exercises require minimal equipment and space, making them well suited to the activity preferences and rehabilitation needs of postpartum women.

### 4.5 Limitations

Several limitations of this study should be acknowledged. The included studies varied in patient characteristics such as time since delivery and incontinence severity, but inconsistent reporting precluded subgroup analyses to assess potential effect modification. A further consideration relates to the methodological quality of the included trials: while only RCTs were included, many had “some concerns” regarding bias due to insufficient reporting of randomization and blinding, which may introduce uncertainty into the estimates. It should also be noted that the predominantly Chinese study population may limit generalizability to other healthcare settings. Additionally, due to the nature of exercise interventions, blinding was often not feasible, and patient-reported outcomes are inherently subjective. Finally, despite Egger’s test suggesting low publication bias (*p* > 0.05), the possibility of unpublished studies cannot be completely excluded. Given these limitations, our findings should be interpreted with caution.

## 5 Conclusion

This network meta-analysis suggests that the selection of exercise interventions for PSUI can be tailored to individual patient goals. PFMT combined with vaginal weights may be preferred for reducing objective leakage, while PFMT with biofeedback and electrical stimulation is most effective for improving pelvic floor muscle strength. Aerobic exercise may offer particular benefits when improvement in quality of life is the primary concern. These findings provide practical guidance for clinical decision-making, allowing clinicians to match intervention type to patient priorities. However, given the limitations identified, these recommendations should be interpreted with caution. Future trials with standardized outcome measures and consistent reporting of patient characteristics are needed to confirm these findings.

## Data Availability

All data generated or analyzed during this study are included in this published article and its Supplementary Files.
