# Supplementary material for: Effects of various exercise interventions for postpartum stress urinary incontinence: a systematic review and network meta-analysis
Source: Front Med (Lausanne). 2026 Apr 30;13:1795125. doi: 10.3389/fmed.2026.1795125 (PMC13171551; doi:10.3389/fmed.2026.1795125)
Supplement: Supplementary file 1 [file Data_Sheet_1.PDF]

Supplementary Material

Effects of various exercise interventions for postpartum stress urinary incontinence: A systematic review and network meta-analysis

Yujia Yin[1],Jiawen Guo[1], Huadong Li[2,*] and Yingjie Qiao [2,*]

[1.] Shandong University of Traditional Chinese Medicine, Shandong, Jinan, 250014, China.

[2.] Department of Tuina, The Affiliated Hospital of Shandong University of Traditional Chinese Medicine, Jinan, 250011, China.

[*] Corresponding author.

E-mail addresses: mhuadong@126.com (HD. Li),648684957@qq.com. (YJ, Qiao).

These authors contributed equally to this work and share first authorship

1 Supplementary file 1

Supplementary file 1. Search strategies for each database.

Supplementary Table 1. Search strategies for each database.

Database name	search strategies
CNKI	SU =('压力性尿失禁' +'尿失禁') AND SU=('产后' + '产后期' + '分娩后') AND SU= ('运动疗法' + '盆底肌训练' + '凯格尔运动' + '骨盆底运动' + '物理治疗' + '康复训练' + '瑜伽' + '普拉提' + '太极' + '太极拳' + '生物反馈' + 呼吸训练') AND FT='随机'
Wanfang	主题: (“压力性尿失禁” OR“尿失禁”) AND 主题:(“产后” OR “产后期” OR “分娩后”) AND 主题: (“运动疗法” OR “盆底肌训练” OR “凯格尔运动” OR “骨盆底运动” OR “物理治疗” OR “康复训练” OR “瑜伽” OR “普拉提” OR “太极” OR “太极拳” OR “生物反馈” OR 呼吸训练”) AND 全部:(随机)
VIP	(M=(压力性尿失禁 OR 尿失禁) OR K=(压力性尿失禁 OR 尿失禁)) AND (K=(产后 OR 产后期 OR 分娩后) AND (产后 OR 产后期 OR 分娩后)) AND M=(运动疗法 OR 盆底肌训练 OR 凯格尔运动 OR 骨盆底运动 OR 物理治疗 OR 康复训练 OR 瑜伽 OR 普拉提 OR 太极 OR 太极拳 OR 生物反馈 OR 呼吸训练) OR K=(运动疗法 OR 盆底肌训练 OR 凯格尔运动 OR 骨盆底运动 OR 物理治疗 OR 康复训练 OR 瑜伽 OR 普拉提 OR 太极 OR 太极拳 OR 生物反馈 OR 呼吸训练)) AND U=随机
Sinomed	#1 "运动疗法"[不加权:扩展]<br>#2 "尿失禁, 压力性"[不加权:扩展]<br>#3 (""产后""[常用字段:智能] OR ""产褥期""[常用字段:智能] OR ""产后""[常用字段:智能] OR ""产褥期""[常用字段:智能]) AND(""压力性尿失禁""[常用字段:智能] OR ""尿失禁""[常用字段:智能] OR ""SUI""[常用字段:智能])<br>#4 (""运动疗法""[常用字段:智能] OR ""锻炼""[常用字段:智能] OR ""训练""[常用字段:智能] OR ""康复""[常用字段:智能] OR ""凯格尔

	""[常用字段:智能] OR ""Kegel""[常用字段:智能] OR " “盆底肌” "[常用字段:智能] OR "” 电刺激” "[常用字段:智能] OR " “生物反馈” "[常用字段:智能] OR " “瑜伽” "[常用字段:智能] OR " “普拉提” "[常用字段:智能] OR " “中国传统功法” "[常用字段:智能] OR " “运动” "[常用字段:智能])<br>#5 ("("随机""[全部字段:智能] OR ""随机对照""[全部字段:智能])<br>#6 #1 OR #4<br>#7 #2 OR #3<br>#8 #7 AND #6 AND #5
Pubmed	#1 ("Postpartum Period"[Mesh] OR postpart* OR postnatal* OR "after birth" OR puerperium)<br>#2 ("Urinary Incontinence, Stress"[Mesh] OR "stress urinary incontinence" OR SUI)<br>#3 ("Exercise Therapy"[Mesh] OR "Pelvic Floor"[Mesh] OR exercis* OR train* OR rehab* OR "Kegel" OR "pelvic floor muscle" OR biofeedback OR electromyography OR "electric stimulation" OR yoga OR pilates OR tai ji OR "core train*")<br>#4 ("random*")<br>#5 #1 AND #2 AND #3AND #4
Web Of Science	TS=("Stress Urinary Incontinence" OR "Urinary Incontinence, Stress" OR "Stress Incontinence" OR SUI) AND TS=(postpartum OR "post natal" OR "post-natal" OR postnatal OR "after birth" OR "after delivery" OR puerperium OR postpart*)AND TS=("Exercise Therapy" OR "Pelvic Floor" OR "Kegel Exercis*" OR "Pelvic Floor Muscle Training" OR PFMT OR exercis* OR "Physical Therapy" OR physiotherapy OR biofeedback OR yoga OR pilates OR "tai ji" OR "tai chi" OR "Breathing Exercises") AND TS= ("random*")
Embase	#1 'puerperium'/exp OR 'post partum care':ab,ti OR 'post partum period':ab,ti OR 'postpartum care':ab,ti OR 'postpartum period':ab,ti OR 'puerperal':ab,ti OR 'puerperal care':ab,ti OR 'puerperium':ab,ti<br>#2 stress AND 'incontinence'/exp OR 'incontinence, stress':ab,ti OR 'stress urinary incontinence':ab,ti OR 'stress urine incontinence':ab,ti OR 'urinary incontinence, stress':ab,ti OR 'urinary stress incontinence':ab,ti OR 'urine stress incontinence':ab,ti OR 'stress incontinence':ab,ti<br>#3 'kinesiotherapy'/exp OR 'corrective exercise':ab,ti OR 'exercise movement techniques':ab,ti OR 'exercise therapy':ab,ti OR 'exercise treatment':ab,ti OR 'kinesiotherapeutic intervention':ab,ti OR 'kinesiotherapeutic method':ab,ti OR 'kinesiotherapeutic procedure':ab,ti OR 'kinesiotherapeutic technique':ab,ti OR 'kinesiotherapeutical treatment':ab,ti OR 'kinesitherapeutic exercises':ab,ti OR 'kinesitherapeutic intervention':ab,ti OR 'kinesitherapeutic method':ab,ti OR 'kinesitherapeutic methodology':ab,ti OR 'kinesitherapeutic procedure':ab,ti OR 'kinesitherapeutic technique':ab,ti OR 'kinesitherapeutic treatment':ab,ti OR 'kinesitherapeutical treatment':ab,ti

	OR 'kinesitherapy':ab,ti OR 'sktm (specialized kinesitherapeutic methodology)':ab,ti OR 'specialised kinesitherapeutic methodology':ab,ti OR 'specialized kinesitherapeutic methodology':ab,ti OR 'therapeutic exercise':ab,ti OR 'therapy, exercise':ab,ti OR 'treatment, exercise':ab,ti OR 'kinesiotherapy':ab,ti<br>#4 #1 AND #2 AND #3
Cochrane Library	#1 MeSH descriptor: [Postpartum Period] explode all trees<br>#2 MeSH descriptor: [Urinary Incontinence, Stress] explode all trees<br>#3 MeSH descriptor: [Exercise Therapy] explode all trees<br>#4 'incontinence, stress' OR 'stress urinary incontinence' OR 'stress urine incontinence' OR 'urinary incontinence, stress' OR 'urinary stress incontinence' OR 'urine stress incontinence' OR 'stress incontinence'<br>#5 'post partum care' OR 'post partum period' OR 'postpartum care' OR 'postpartum period' OR 'puerperal' OR 'puerperal care' OR 'puerperium'<br>#6 'incontinence, stress' OR 'stress urinary incontinence' OR 'stress urine incontinence' OR 'urinary incontinence, stress' OR 'urinary stress incontinence' OR 'urine stress incontinence' OR 'stress incontinence'<br>#7 #1 OR #5<br>#8 #2 OR #4<br>#9 #3 OR #6<br>#10 #7 AND #8 AND #9

Abbreviations: CNKI, China National Knowledge Infrastructure; SinoMed, Chinese Biomedical Literature Database; WanFang, WanFang Database; VIP, Chinese Scientific Journals Full-Text Database.

2 Supplementary file 2

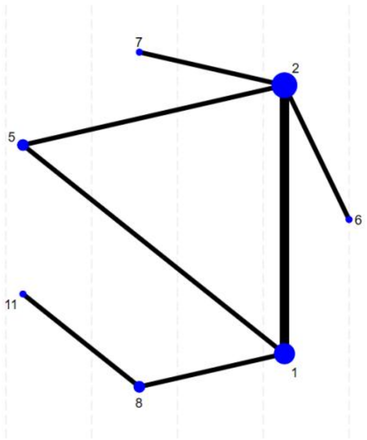

Supplementary Fig. 2. The network plot for secondary psychosocial outcomes.

3 Supplementary file 3

Forest plots from the network meta-analysis of other indicators.

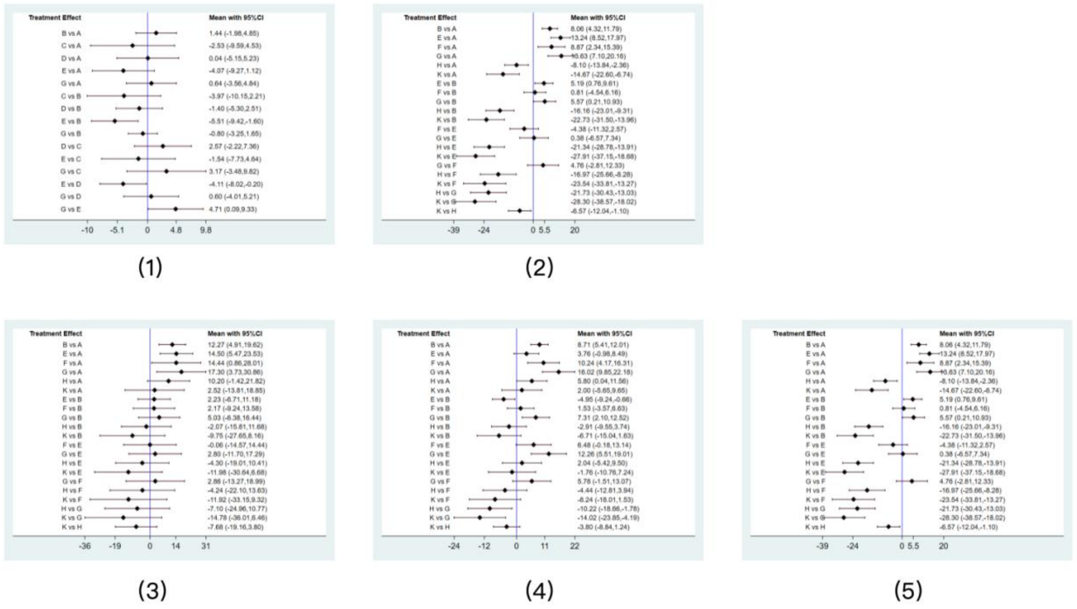

Supplementary Fig. 3. Forest plots for secondary outcomes. (1) 1-Hour Pad Test; (2) ICIQ-SF Scores; (3) Psychological factors; (4) Social barriers; (5) Behavioral limitations.

4 Supplementary file 4

SUCRA ranking plots from the network meta-analysis.

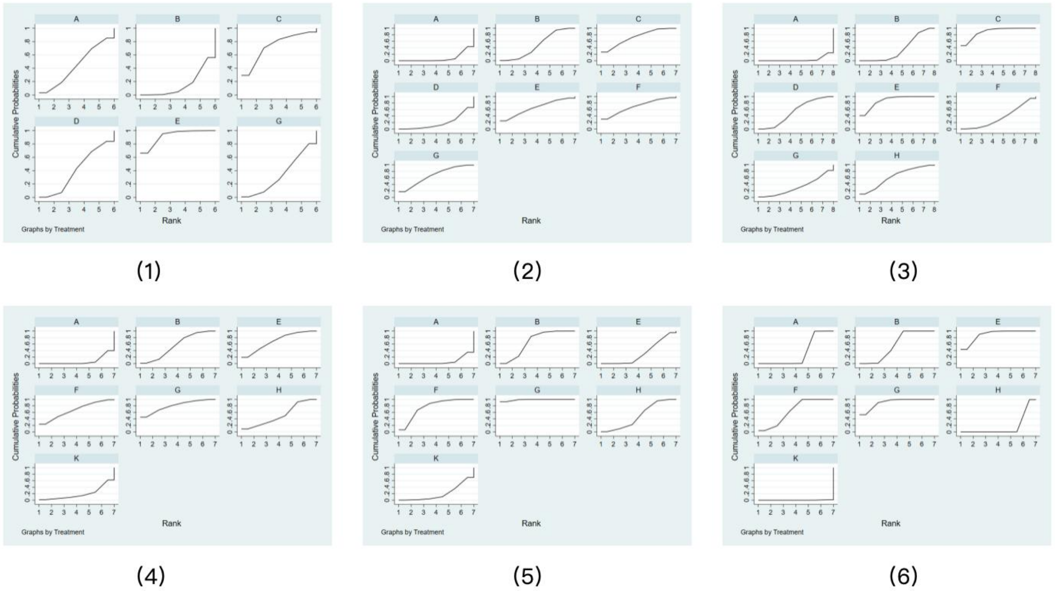

Supplementary Fig. 4. SUCRA ranking plots for all outcomes. (1) 1-Hour Pad Test; (2) ICIQ-SF Scores; (3) Pelvic floor muscle strength improvement; (4) Psychological factors; (5) Social barriers; (6) Behavioral limitations.

5 Supplementary file 5

League tables from the network meta-analysis of secondary outcomes.

(1)

C							
0.09 (-2.28,2.46)	E						
1.32 (-1.51,4.15)	1.23 (-1.74,4.20)	H					
1.75 (-0.34,3.85)	1.66 (-0.15,3.47)	0.43 (-2.38,3.24)	D				
2.47 (0.78,4.16)	2.38 (0.46,4.30)	1.15 (-1.12,3.42)	0.72 (-0.95,2.38)	B			
2.57 (-0.03,5.17)	2.48 (-0.05,5.00)	1.25 (-1.83,4.32)	0.82 (-1.72,3.36)	0.10 (-1.97,2.17)	F		
2.80 (-0.31,5.91)	2.70 (-0.54,5.94)	1.47 (-1.99,4.93)	1.04 (-2.05,4.14)	0.33 (-2.29,2.94)	0.23 (-3.11,3.56)	G	
4.06 (2.14,5.99)	3.97 (2.15,5.79)	2.74 (0.26,5.22)	2.31 (0.53,4.09)	1.59 (0.60,2.59)	1.50 (-0.44,3.43)	1.27 (-1.53,4.06)	A

(2)

G						
2.76 (-11.67,17.19)	E					
2.88 (-13.18,18.94)	0.12 (-14.31,14.54)	F				
5.01 (-6.34,16.37)	2.25 (-6.66,11.16)	2.14 (-9.22,13.49)	B			
7.04 (-10.74,24.81)	4.27 (-10.37,18.92)	4.16 (-13.61,21.93)	2.02 (-11.66,15.70)	H		
14.72 (-6.41,35.85)	11.96 (-6.62,30.53)	11.84 (-9.28,32.97)	9.71 (-8.11,27.53)	7.68 (-3.75,19.11)	K	
17.23 (3.73,30.73)	14.47 (5.48,23.46)	14.35 (0.86,27.85)	12.21 (4.91,19.52)	10.19 (-1.37,21.75)	2.51 (-13.74,18.76)	A

(3)

G						
12.26 (5.54,18.98)	E					
5.78 (-1.47,13.04)	-6.48 (-13.12,0.16)	F				
7.31 (2.12,12.49)	-4.95 (-9.23,-0.68)	1.52 (-3.55,6.60)	B			
10.20 (1.80,18.61)	-2.06 (-9.49,5.37)	4.42 (-3.92,12.76)	2.90 (-3.72,9.51)	H		
14.01 (4.22,23.79)	1.75 (-7.22,10.71)	8.22 (-1.50,17.95)	6.70 (-1.60,15.00)	3.80 (-1.21,8.82)	K	
16.00 (9.86,22.14)	3.74 (-0.97,8.45)	10.22 (4.17,16.26)	8.69 (5.41,11.98)	5.79 (0.05,11.54)	1.99 (-5.63,9.61)	A

(4)

G						
0.38 (-6.56,7.32)	E					
4.76 (-2.80,12.33)	4.38 (-2.55,11.32)	F				
5.57 (0.21,10.92)	5.19 (0.77,9.61)	0.81 (-4.54,6.15)	B			
21.71 (13.02,30.40)	21.33 (13.90,28.76)	16.95 (8.27,25.63)	16.14 (9.30,22.99)	H		
28.28 (18.02,38.54)	27.90 (18.68,37.12)	23.52 (13.26,33.78)	22.71 (13.96,31.47)	6.57 (1.10,12.03)	K	
13.62 (7.09,20.14)	13.24 (8.51,17.96)	8.85 (2.34,15.37)	8.05 (4.32,11.78)	-8.10 (-13.83,-2.36)	-14.66 (-22.59,-6.74)	A

Supplementary Fig. 5. League tables for secondary outcomes. (1) Pelvic floor muscle strength improvement; (2) Psychological factors; (3) Social barriers; (4) Behavioral limitations.

6 Supplementary file 6

Results of Egger's test and the corresponding funnel plot for primary outcomes.

(1)

```
. metabias _y _stderr, egger graph

Note: data input format theta se_theta assumed

Egger's test for small-study effects:
Regress standard normal deviate of intervention
  effect estimate against its standard error

.
Number of studies =  10                        Root MSE      =    14.59
------------------------------------------------------------------------
     Std_Eff     Coef.   Std. Err.      t    P>|t|     [95% Conf. Interval]
------------------------------------------------------------------------
       slope     0.520      0.884     0.59   0.573     -1.518       2.557
        bias    -5.260      6.577    -0.80   0.447    -20.426       9.907
------------------------------------------------------------------------

Test of H0: no small-study effects          P = 0.447
```

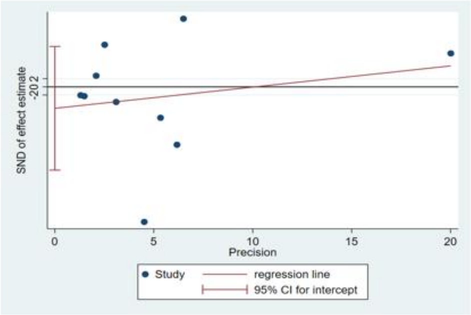

(2)

```
. metabias _y _stderr, egger graph

Note: data input format theta se_theta assumed

Egger's test for small-study effects:
Regress standard normal deviate of intervention
  effect estimate against its standard error

.
Number of studies =  14                        Root MSE      =    15.26
------------------------------------------------------------------------
     Std_Eff     Coef.   Std. Err.      t    P>|t|     [95% Conf. Interval]
------------------------------------------------------------------------
       slope     0.754      2.008     0.38   0.714     -3.620       5.129
        bias    -7.149      8.130    -0.88   0.396    -24.862      10.564
------------------------------------------------------------------------

Test of H0: no small-study effects          P = 0.396
```

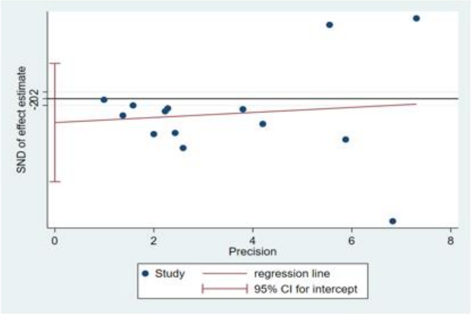

Supplementary Fig. 6. Egger's test funnel plots for primary outcomes. (1) 1-Hour Pad Test (P = 0.447); (2) ICIQ-SF Scores (P = 0.396).

7 Supplementary file 7

Funnel plots for secondary outcomes.

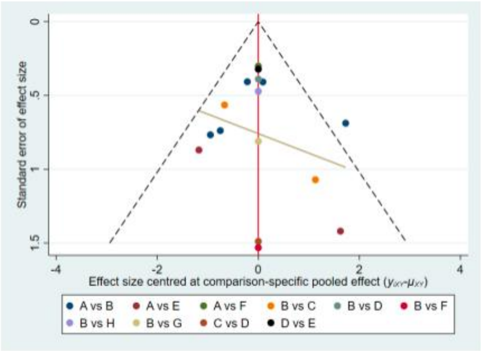

(1)

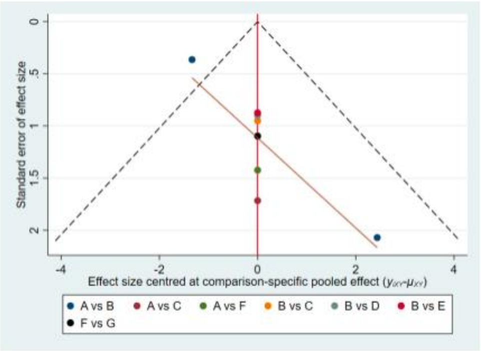

(2)

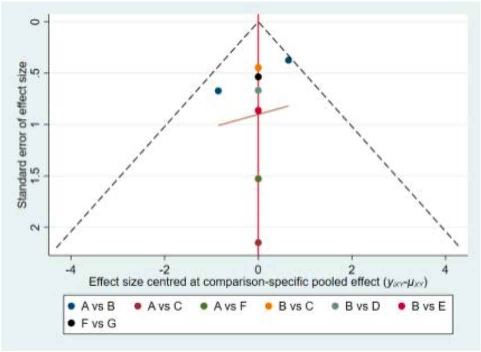

(3)

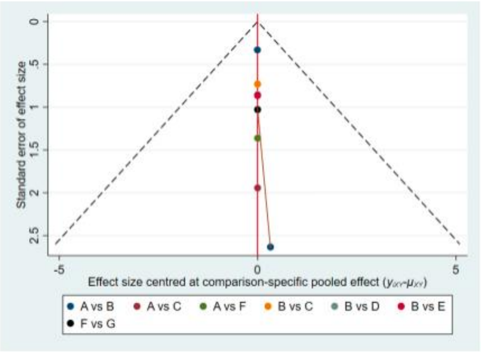

(4)

Supplementary Fig. 7. Funnel plots for secondary outcomes. (1) Pelvic floor muscle strength improvement; (2) Psychological factors; (3) Social barriers; (4) Behavioral limitations.
